# Plants Recruit Peptides and Micro RNAs to Regulate Nutrient Acquisition from Soil and Symbiosis

**DOI:** 10.3390/plants12010187

**Published:** 2023-01-02

**Authors:** Marios I. Valmas, Moritz Sexauer, Katharina Markmann, Daniela Tsikou

**Affiliations:** 1 Department of Biochemistry and Biotechnology, University of Thessaly, Biopolis, 41500 Larissa, Greece; 2 Julius-von-Sachs-Institute for Biosciences, Würzburg University, Julius-von-Sachs-Platz 3, 97082 Würzburg, Germany

**Keywords:** CEP/CLE peptide hormones, mobile miRNAs, nutrient homeostasis, root symbiosis

## Abstract

Plants engage in symbiotic relationships with soil microorganisms to overcome nutrient limitations in their environment. Among the best studied endosymbiotic interactions in plants are those with arbuscular mycorrhizal (AM) fungi and N-fixing bacteria called rhizobia. The mechanisms regulating plant nutrient homeostasis and acquisition involve small mobile molecules such as peptides and micro RNAs (miRNAs). A large number of CLE (CLAVATA3/EMBRYO SURROUNDING REGION-RELATED) and CEP (C-TERMINALLY ENCODED PEPTIDE) peptide hormones as well as certain miRNAs have been reported to differentially respond to the availability of essential nutrients such as nitrogen (N) and phosphorus (P). Interestingly, a partially overlapping pool of these molecules is involved in plant responses to root colonization by rhizobia and AM fungi, as well as mineral nutrition. The crosstalk between root endosymbiosis and nutrient availability has been subject of intense investigations, and new insights in locally or systemically mobile molecules in nutrient- as well as symbiosis-related signaling continue to arise. Focusing on the key roles of peptides and miRNAs, we review the mechanisms that shape plant responses to nutrient limitation and regulate the establishment of symbiotic associations with beneficial soil microorganisms.

## 1. Introduction

Plant growth and development depend on the acquisition of a number of mineral nutrients from the soil. Essential nutrients such as nitrogen (N) and phosphorus (P) have key roles in agriculture, as their limitation is considered a frequent cause of reduced crop productivity. Most land plants meet nutrient limitation in terrestrial environments by associating with beneficial microorganisms. Arbuscular mycorrhizal (AM) associations with fungi and N-fixing root nodulation of legume plants with rhizobial bacteria improve the acquisition of mineral elements, such as P and N. Microbial inoculants are increasingly used as biofertilizers, and tested for their potential to replace cost-intensive and environmentally harmful synthetic P and N fertilizers in agricultural settings.

This review discusses the role of peptides and micro RNAs (miRNAs) in mediating the plant responses to N and P availability and the establishment and control of symbiotic relationships improving N and P acquisition.

## 2. Plants Associate with Soil Microorganisms to Access Essential Nutrients

Root nodule symbiosis is an endosymbiotic association formed between legumes and rhizobial bacteria. Under symbiotic conditions, the latter fix aerial N_2_ through the enzyme nitrogenase, converting it to ammonia (NH_3_) (reviewed in [1]). Upon release to the peribacteroid space that separates symbiotic bacteria from the infected host cell, NH_3_ is converted to ammonium (NH_4_^+^), which is then released to the plant cytosol [2]. NH_4_^+^ transporters have been characterized in legumes, as in soybean (*Glycine max*) [3] and *Lotus japonicus* [4]. In return for fixed N, plants provide rhizobia with branched amino acids, sugars and micronutrients essential for bacterial development. Besides that, dicarboxylic acids, mainly malate, are also provided to bacteria by the plant, and are essential for N fixation [1].

During the establishment of the symbiotic relationship, communication signals are exchanged between rhizobia and legumes, involving flavonoids, which are released into the rhizosphere by the plant root [5,6] and trigger the production of lipochitooligosaccharide (LCO) nodulation factors (Nod factors) in compatible rhizobia (reviewed in [7]). Nod factor signaling triggers a response cascade resulting in rhizobial entry into the root epidermis and cortex, paralleled by the formation of a nodule primordium. Nodules are lateral root organs where rhizobia are hosted intracellularly and develop into N-fixing bacteroids surrounded by a plant-derived membrane individually or in small groups, forming organelle-like symbiosomes (reviewed in [8]).

Arbuscular mycorrhizal symbiosis, the association formed between plants and fungi of the phylum Glomeromycota, plays a critical role in nutrient acquisition by providing access predominantly to P, but also to N and other mineral nutrients. AM fungi were found to possess high-affinity transporters of inorganic phosphate (P) [9], which accumulates as polyphosphate within arbuscules and is then rapidly translocated to the host plant [10]. N is also taken up by AM fungi from the substrate, and genes involved in the transfer of NH_4_^+^ and amino acids to host plants have been identified [11,12]. AM fungi receive photosynthetic carbon in the form of sugars and lipids (reviewed in [13]) and are obligate biotrophs, strictly relying on host plant resources for growth and reproduction.

Early chemical communication between AM fungi and host plants involves strigolactones released by plant roots [14], and a cocktail of fungal chitooligosaccharides (COs) and lipochitooligosaccharides (LCOs) [15,16]. AM fungal entry into the root is achieved through appressoria that develop on the root epidermal surface [17]. Following hyphal entry, highly branched fungal arbuscules are formed within cells of the inner root cortex. Like symbiosomes in nodules, these are surrounded by a plant plasmalemma-derived membrane and represent the major sites of nutrient exchange between micro- and macrosymbiont [18]. Arbuscules have a limited lifetime, and following their collapse and digestion by the host cell, the latter can be re-colonized by a new arbuscule (reviewed in [19]).

## 3. Plant Responses to N Availability and Rhizobial Symbiosis Involve CEP and CLE Peptide Regulation

Peptide hormones facilitate both cell-to-cell signaling in plant tissues and systemic communication between organs by long-distance mobility through the vascular system. Plant genomes encode a variety of small signaling peptides (SSPs), which in their mature state are post-translationally modified, small (<20 amino acids) peptides cleaved from a longer precursor protein, and are involved in developmental and physiological processes and mediating plant responses to environmental stimuli. Several SSP gene families show differential abundances in response to changes in plant nutrient status, and have roles in processes controlling root morphogenesis and physiology, as well as macronutrient uptake [20,21]. The CLE (CLAVATA3/EMBRYO SURROUNDING REGION-RELATED) and CEP (C-TERMINALLY ENCODED PEPTIDES) families have been studied extensively in relation to their roles in systemic N signaling. Members of other SSP gene families including CAPE (CAP-DERIVED PEPTIDE), GLV *(*GOLVEN/ROOT GROWTH FACTOR), IDA (INFLORESCENCE DEFICIENT IN ABCISSION), PIP (PAMP-INDUCED SECRETED PEPTIDE) and TAX (TAXIMIN) encoding genes were similarly suggested to play roles in nutrient-status-related signaling [20]. In the following paragraphs we discuss the roles of CLE and CEP peptides in N deficiency and nodulation symbiosis signaling.

### 3.1. Roles of CLE Peptides in N Homeostasis and Symbiosis Regulation

CLE peptides are 12 to 13 amino acids long and function as secreted peptide ligands that bind to plasma membrane-localized receptor-like proteins, thereby triggering downstream signaling events. The CLE gene family encodes small proteins with a conserved CLE domain at the C-terminus, generating the mature CLE peptide following proteolytic processing [22]. CLE peptides regulate various physiological and developmental processes, and a number of CLEs were reported to be involved in nutrient homeostasis and to respond to symbiotic interactions with microorganisms [23].

The *Arabidopsis thaliana* genome harbors 32 *CLE* genes [22]. Among them, *CLE1*, *-3*, *-4* and *-7* show increased activity in N-deficient compared to sufficient roots and were suggested to regulate lateral root primordia formation through binding to the CLAVATA1 (CLV1) leucine-rich repeat-receptor-like kinase [24]. These CLE genes are expressed in the root pericycle, and the corresponding CLE peptides are hypothesized to be secreted from pericycle cells and transported through the apoplastic continuum within the central cylinder to reach phloem companion cells where CLV1 is localized. The CLE-CLV1 signaling pathway is a key mechanism regulating the outgrowth of lateral roots and the expansion of the root system when *A. thaliana* plants grow under N-deficient conditions, enhancing the plant survival in N-poor environments [24].

**Figure 1:**
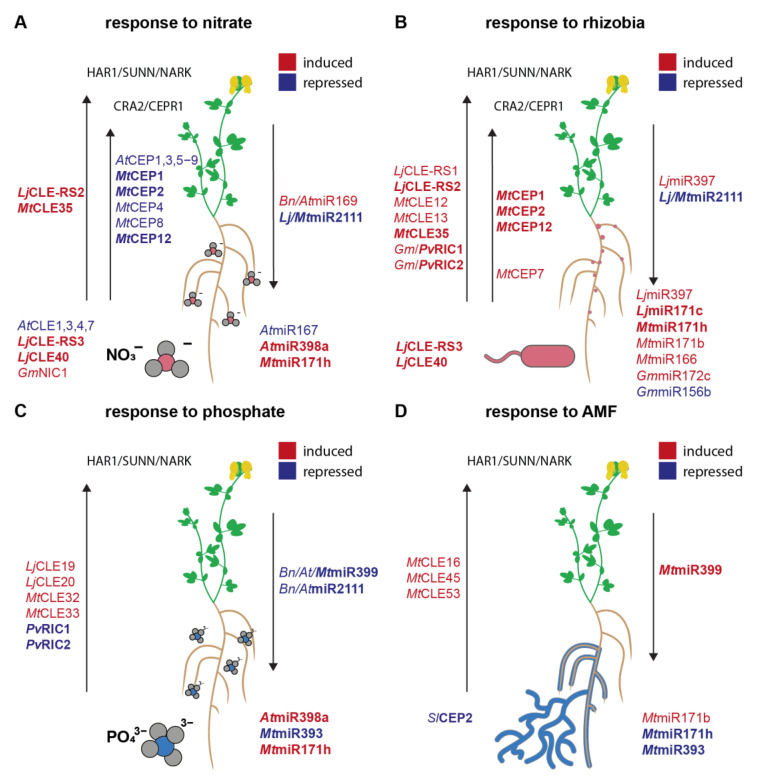
Nutrient homeostasis and acquisition mechanisms involve regulation by peptide hormones and miRNAs. CLE and CEP peptides and miRNAs responding to (**A**) N availability, (**B**) rhizobia , (**C**) P availability and (**D**) arbuscular mycorrhizal fungi. Molecules that are induced or repressed by a respective stimulus are displayed in red or blue, respectively. Molecules that are responsive to more than one stimulus are in bold. Arrows indicate shoot-to-root or root-to-shoot translocation of mobile molecules. Specific responses are mediated by the shoot localized leucine-rich repeat receptor-like kinases HAR1/SUNN/NARK and CRA2/CEPR1. *Lj*, *Lotus japonicus*; *Mt*, *Medicago truncatula*; *At*, *A. thaliana*; *Bn*, *Brassica napus*; *Gm*, *Glycine max*; *Pv*, *Phaseolus vulgaris*; *Sl*, *Solanum lycopersicum*.

In legume plants, multiple *CLE* genes have been proposed to be involved in nodulation control. Some legume *CLE* genes are specifically linked to the rhizobial symbiosis, while others are regulated by both rhizobia and N availability. A number of CLE peptides have been reported to negatively regulate nodulation, acting as essential components of a plant mechanism called autoregulation of nodulation (AON) which balances nodule numbers with plant needs and resource availability (for a recent review see [25]). *CLE* genes related to rhizobial infection or symbiosis include *L. japonicus LjCLE-RS1*, *-2*, *-3* and *LjCLE40* [26,27], *M. truncatula MtCLE12*, *-13* and *-35* [28,29,30], *Glycine max GmRIC1* and *-2* [31] and *Phaseolus vulgaris PvRIC1* and *-2* [32] (Figure 1, Table 1). Among them, *LjCLE-RS1*, *MtCLE12*, *MtCLE13*, *GmRIC1* and *GmRIC2* were reported to specifically show increased expression activity in roots upon rhizobial infection compared to mock treated roots [27,28,31]. Consistent with root–shoot mobility, *LjCLE-RS2* derived peptides, though specifically expressed in roots, were found in xylem sap collected from shoot tissue of infected plants [26]. *Lj*CLE-RS2 peptides were further found to directly bind to the shoot-localized CLV1-type leucine-rich repeat receptor-like kinase (LRR-RLK) *Lj*HAR1 (HYPERNODULATION ABERRANT ROOT FORMATION 1) [33], a negative regulator of symbiosis [34,35]. Putative orthologues of *Lj*HAR1 in other legumes, the symbiosis regulators *Mt*SUNN, *Gm*NARK [36] and *Pv*NARK [32] are likely to similarly act as receptors of rhizobia-induced, xylem-mobile CLE peptides.

In a process analogous to AON, root nodulation symbiosis is inhibited by high nitrate concentrations in the environment. *LjCLE-RS2* expression is induced by both rhizobial inoculation and nitrate supply, implying a dual role in rhizobia-induced autoregulation and nitrate-mediated inhibition of nodulation [26]. Studies on CLE35 in *M. truncatula* offer further evidence for an involvement of AON components in nitrate inhibition of nodulation. *MtCLE35* is a nitrate-responsive gene, which is also expressed during nodulation [29]. Overexpression of *MtCLE35* in transgenic roots of *M. truncatula* led to reduced root nodule numbers, in a *SUNN*- dependent manner [30]. Additionally, downregulation of *MtCLE35* through RNAi resulted in increased nodule numbers, even under nitrate conditions where nodulation was inhibited in wild-type plants [37]. *MtCLE34* was also co-induced by nitrate and rhizobia but turned out to be a pseudogene lacking a functional CLE domain [30]. It was thus proposed that *MtCLE34* might have had a role in nodulation, before it was mutated and lost its function [30].

Biochemical studies revealed that CLE peptides are post-translationally modified. In the well-studied CLV3 peptide of *A. thaliana*, a proline residue at position 7 is hydroxylated and subsequently arabinosylated, a prerequisite for its biological activity and high-affinity binding to its receptor CLV1 [38]. Hydroxyproline *O*-arabinosylation is widely observed in secreted *A. thaliana* peptides, and Golgi-localized enzymes encoded by three *AtHPAT* genes mediate this process [39]. CLE arabinosylation was similarly reported in other plants, such as *L. japonicus* [33], *M. truncatula* [40,41] and *P. sativum* [42], suggesting that this modification may be a requirement for receptor binding and functionality in general. In *M. truncatula*, the rhizobium-induced *MtCLE12* was suggested to be arabinosylated by the Golgi-localized hydroxyproline *O*-arabinosyltransferase ROOT DETERMINED NODULATION1 (RDN1), as *MtCLE12* overexpression did not affect root nodule numbers in *rdn1* loss-of-function mutants [40,41]. Interestingly, in contrast to *MtCLE12,* tri-arabinosylation of *MtCLE13* was *RDN1*-independent, suggesting that other enzymes are also involved in CLE peptide arabinosylation in this species [41]. In *L. japonicus*, CLE-RS1 and CLE-RS2 tri-arabinosylation was shown to be critical for HAR1 binding and activity in AON [33]. While the enzyme catalyzing glycosylation of these peptides is unclear, a third CLE mediating HAR1-dependent AON in *L. japonicus*, *Lj*CLE-RS3, was shown to be arabinosylated through *LjPLENTY*, a putative ortholog of *MtRDN1*/*Pisum sativum NOD3,* which are all homologs of *AtHPAT* genes [43]. Consistently, PLENTY also localizes to the Golgi complex. Overexpression of *LjCLE-RS1* and *-2* in a *plenty* mutant background retained AON activity, whereas *LjCLE-RS3* mediated repression of nodulation was abolished in *plenty* mutants [43]. *LjCLE-RS1* and *-2* are thus likely arabinosylated at least in part by enzymes other than PLENTY [43].

**Table 1:** List of selected CLE and CEP peptides responding to nitrogen (N), phosphorus (P) and microsymbionts (rhizobium and AM fungi).

	Stimuli	Organism	Influence Range	Predominant Expression (Tissue)	Refs
*AtCLE1* *AtCLE3* *AtCLE4* *AtCLE7*	N-deficiency induced	*A. thaliana*	systemic	roots	[24]
*LjCLE-RS1*	Rhizobium-induced	*L. japonicus*	systemic	roots	[26]
*LjCLE-RS2*	Rhizobium- and N-induced	*L. japonicus*	local and systemic	roots	[26]
*LjCLE-RS3 LjCLE40*	Rhizobium- and N-induced	*L. japonicus*		roots, nodule primordia	[27]
*LjCLE19 * *LjCLE20*	P-induced	*L. japonicus*		roots	[44]
*MtCLE12*	Rhizobium-induced	*M. truncatula*	local and systemic	nodules	[28]
*MtCLE13*	Rhizobium- and nod factor-induced, cytokinin-induced	*M. truncatula*	local and systemic	roots (symbiosis susceptible zone), inner cortical cells, nodules	[28] [45]
*MtCLE35*	Rhizobium- and N-induced	*M. truncatula*	systemic	roots, nodules	[29] [30]
*MtCLE32*	Pi-induced	*M. truncatula*		roots	[46]
*MtCLE33*	Pi-induced	*M. truncatula*		root vascular tissue	[46] [47]
*MtCLE16 MtCLE45*	AM-induced	*M. truncatula*		roots	[46] [47]
*MtCLE53*	AM-induced	*M. truncatula*		root vascular tissue near colonized regions	[46] [47]
*GmRIC1 * *GmRIC2*	Rhizobium-induced	*G. max*	systemic	roots	[31]
*GmNIC1*	N-induced	*G. max*	local	roots	[31]
*PvRIC1 * *PvRIC2*	Rhizobium-induced, P-deficiency increased	*P. vulgaris*	systemic	roots, pericycle cells of Pi-deficient roots	[32] [48]
*AtCEP1* *AtCEP3* *AtCEP5* *AtCEP6* *AtCEP7* *AtCEP8* *AtCEP9*	N starvation-induced	*A. thaliana*	systemic	mainly roots (but also in aerial tissues)	[49]
*MtCEP1*	Rhizobium-induced, N starvation-induced	*M. truncatula*	local and systemic	roots, shoots	[50,51,52]
*MtCEP2* *MtCEP12*	Rhizobium-induced, N starvation-induced	*M. truncatula*		mainly roots, shoots	[52]
*MtCEP4* *MtCEP5* *MtCEP6* *MtCEP8*	N starvation-induced	*M. truncatula*		mainly roots, shoots	[52]
*MtCEP7*	Rhizobium- and nod factors-induced, cytokinin-induced	*M. truncatula*	systemic	roots, epidermal cells in colonized roots, nodule primordia, mature nodules	[45]
*SlCEP2*	AM-reduced	*S. lycopersicum*	local	roots	[53]

### 3.2. Roles of CEP Peptides in N Homeostasis and Symbiosis Regulation

CEP peptides are a family of SSPs which are 15 amino acids long, secreted peptides released from a C-terminal conserved domain (the CEP domain) of precursor proteins through proteolytic processing. Similarly to CLEs, CEPs are also post-translationally modified by proline hydroxylation and arabinosylation [54]. The accumulation of CEPs was observed to be highly correlated with plant responses to N starvation. The *A. thaliana* genome includes 11 *CEP* genes, 7 of which have been shown to be up-regulated specifically in response to N starvation [49]. Moreover, 10 out of the 11 *CEP* genes led to enhanced expression of the nitrate transporter gene *NRT2.1* when overexpressed in *A. thaliana* seedlings [49].

The well-studied *At*CEP1 peptide in *A. thaliana* was shown to undergo long-distance root-to-shoot translocation and proposed to mediate plant adaptations to low environmental N availability [49]. CEP1 directly binds to the leucine-rich repeat receptor kinases CEPR1 and CEPR2, found to locate in both shoots and roots [49]. The systemic nature of this mechanism was shown via grafting (*cepr1-1 cepr2-1* mutant scions were grafted onto wild-type rootstocks by hypocotyl-to-hypocotyl grafting) and split root (the root system of a plant was separated into two parts exposed to different nutrient conditions) studies, and the translocation of CEP1 was verified by its detection in the xylem sap [49]. Exogenous application of CEP1 and CEPR1/2 loss of function studies showed that the CEP1-CEPR1/2 signaling pathway regulates N uptake by affecting the expression of genes encoding for the nitrate transporters NRT1.1, NRT2.1 and NRT3.1 [49].

Similarly to CLE peptides, CEP peptides have been reported to be involved not only in N-deficiency responses but also nodulation control in legumes. In contrast to the repressive role of CLE peptides on symbiotic nodule numbers, CEPs have been attributed a positive regulatory role in nodulation. *Mt*CEP1 in *M. truncatula* was shown to enhance nodulation when overexpressed or externally applied to *Sinorhizobium meliloti*-infected roots [50]. Exogenous application of *Mt*CEP1 to *M. truncatula* roots led to significantly decreased lateral root numbers, while nodule numbers increased [50]. Both effects were mediated by the LRR-RLK CRA2 (COMPACT ROOT ARCHITECTURE 2)*,* the putative orthologue of *At*CEPR1 in *M. truncatula*, as they were abolished in *cra2* loss-of-function mutants [51]. In addition to *MtCEP1*, *MtCEP2* and *MtCEP12* were N-starvation induced, and co-regulated lateral root and nodule numbers [52] (Table 1). Grafting studies revealed that the CRA2-mediated signaling pathway affecting root architecture is locally active in roots, whereas CRA2-mediated nodulation control is an independent process which is systemically regulated through shoot-localized CRA2 [55]. The systemic *Mt*CEP1-CRA2 node promotes nodulation under low N conditions by regulating the downstream signaling components miR2111 and *TML* (see below) [56].

*Mt*CEP7, which was reported to be induced by rhizobia, Nod factors and cytokinin [45], seems to function as positive regulators of symbiosis, as exogenous CEP7 application reinforced nodulation, whereas *CEP7* downregulation led to reduced nodule numbers [45]. Similarly to *Mt*CEP1, *Mt*CEP7 was also seen to control nodulation through a systemic signaling pathway mediated by the shoot-localized population of the CRA2 receptor [45].

In summary, downstream effects of rhizobium or nitrate-induced CLE and CEP peptides are antagonistic, with CLE peptides mediating restriction, and CEP peptides promoting nodulation. These opposite responses are mediated by partially overlapping signaling pathways sharing common components. Chromatin immunoprecipitation studies revealed that the transcription factor NIN co-regulates the expression of *MtCLE13* and *MtCEP7*, and ectopic expression of *MtNIN* induced the expression of *MtCLE13* and *MtCEP7* in the absence of external stimuli [45]. Moreover, both *MtCLE13* and *MtCEP7* were induced by cytokinin, and the effects of both peptides on nodulation were mediated by the cytokinin receptor gene *MtCRE1* [45]. Studies on the crosstalk between peptide and classical hormones provide evidence that peptide signaling is interlinked with signaling through cytokinin, auxin, ethylene and strigolactones (for a recent review see [57]). The concurrent induction of the antagonistic CLE and CEP pathways may be part of a mechanism that enables the plant to flexibly adjust rhizobial infection events and the nodule numbers to its needs based on the endogenous supply status of various nutrients, photosynthetic capacity and environmental conditions.

## 4. CLEs and CEPs Respond to Both P and AM Fungal Infection

In contrast to root nodulation symbiosis, where host plants are supplied with bacterially fixed aerial N, AM fungi predominantly deliver phosphate extracted from the surrounding substrate to the host. It was shown that high exogenous phosphate supply restricts the initiation and development of AM symbiosis. P acts systemically to repress symbiotic gene expression and AM fungal root colonization [58].

Analogous to CLE-mediated regulation of nodulation symbiosis, this regulation of AM involves CLE peptides (Table 1, Figure 1). In *M. truncatula,* expression of *MtCLE32* and *MtCLE33* was significantly induced in roots grown under high (2 mM) P conditions compared to P-starved roots [46]. Further, ectopic overexpression of the *MtCLE33* in *M. truncatula* transgenic roots resulted in reduced AM root colonization [46]. Apart from peptides, phytohormones and miRNAs have been reported to have key roles in P starvation and AM symbiosis signaling, regulating the initiation, maintenance, and extent of AM root colonization (reviewed in [59]).

The development of AM fungi within the root is regulated by the host plant through a genetic mechanism termed autoregulation of mycorrhizal symbiosis (AOM) [60], a systemic signaling cascade sharing common elements with AON [61]. Along this line, it was shown that root-derived CLE peptides and a CLV1-type shoot-localized receptor regulate the colonization of roots by AM fungi [46,47]. Transcript abundance of specific *CLE* genes was found to increase upon AM fungal root colonization [46,47,62]. Certain *CLE* genes responding to AM symbiosis were also shown to respond to phosphate availability [47] (Table 1, Figure 1), reminiscent of the dual regulation of *CLE* genes by rhizobial infection and nitrate [26,27]. Ectopic overexpression of the AM-induced *MtCLE53* in the roots of *M. truncatula* resulted in reduced fungal colonization compared to control roots [46,47], whereas *cle53* mutants showed higher colonization levels than wild-type plants [47]. Interestingly, the nodulation-induced *MtCLE13* [28] was not induced by AM symbiosis, and ectopic overexpression of *MtCLE13* seems not to have an effect on fungal colonization levels, implying specificity of the respective *CLEs* [46].

Similarly to AON, arabinosylation of CLE peptides may also be a requirement for receptor binding and functionality in AOM. Karlo et al. [47] showed that the hydroxyproline *O*-arabinosyltransferase RDN1 has a role in the control of fungal colonization in *M. truncatula*. Mycorrhized roots of *rdn1* mutants contained more vesicles and arbuscules than wild-type roots. In line with a requirement for RDN1-mediated arabinosylation of *MtCLE53*, overexpression of the latter in an *rdn1* genetic background did not reduce AM fungal colonization as in wild-type plants [47].

Although CLE peptides may respond to diverse stimuli, the shoot-localized receptor *Lj*HAR1/*Mt*SUNN/*Gm*NARK/*Ps*SYM29, may be a common component of the respective signaling mechanisms (discussed in [57]). In *M. truncatula*, downstream signaling of the AM-induced *MtCLE53*, the rhizobium-induced *MtCLE13* or the P-responsive *MtCLE33* was dependent on SUNN in overexpression assays, implying SUNN as a common receptor for all three CLE peptides [45,46]. Components acting downstream of the shoot receptor in AOM are still unknown, except that it was shown that the control of fungal root colonization in *M. truncatula* seems mediated by regulation of strigolactone biosynthesis via *M. truncatula DWARF27* (*MtD27*) expression [63]. This regulation was shown to be dependent on P levels and AM signaling, and was mediated by SUNN and CLEs [46].

In addition to CLE peptides, a genome-wide investigation of SSPs in *M. truncatula* found CEPs to be responsive to P deficiency [21]. Further, recent findings showed *CEP2* to be downregulated in AM-inoculated *S. lycopersicum* roots. *Sl*CEP2 was proposed to promote lateral root formation in tomato plants through an auxin-related pathway, which might be CEPR1*-*mediated [53]. However, so far, no direct evidence of a functional involvement of CEPs in AOM has been reported, and a putative function in AM control will be an interesting subject of future studies.

## 5. miRNAs Respond to N and P Availability and Symbiosis-Mediated Nutrient Acquisition

MicroRNAs are small, non-coding RNA molecules, typically 21–24 nucleotides in length, that exert post-transcriptional gene regulation by homology-based pairing to target mRNAs, inducing their degradation or translational inhibition.

Several miRNAs have been associated with responses to N availability in different plant species (Table 2, Figure 1). In *A. thaliana*, upon N starvation, the expression of one or more miRNAs of the miR169, miR171 and miR395 families was repressed, while miR160 and miR780 expression was induced [64]. In addition, an *A. thaliana* miR167 isoform was the first miRNA to be linked to plant N-responses, and was shown to mediate N dependent lateral root outgrowth [65]. More studies in *A. thaliana*, but also other plants, have shown that the regulation of the plant root architecture is a major function of N-responsive miRNAs. Interestingly, both miR167 and miR393 influence root architecture by interfering with auxin signaling through targeting the AUXIN RESPONSE FACTOR 8 (ARF8) [65] and the AUXIN-SIGNALING F-BOX PROTEIN 3 (AFB3) [66], respectively. Further, miR169 targets the transcript of *NFYA5*, which encodes a transcription factor suggested to regulate N-starvation responses in plants by affecting the expression of the nitrate transporters *AtNRT1.1* and *AtNRT2.1* [67]. Interestingly, apart from miRNAs, also long non-coding RNAs (lncRNAs) have been found to respond to the N status in different plants (for a recent review see [68]).

Consistent with a general role of miRNAs in maintaining plant nutrient homeostasis, several miRNAs have further been reported to respond to P availability (Table 2, Figure 1). Among them, miR399, miR827 and miR2111 isoforms were found to accumulate under P-starvation conditions in different plant species including *A. thaliana* and *N. benthamiana*. These miRNAs were present in the phloem sap of P-starved *B. napus* plants, suggesting organ-to-organ mobility along with long-distance regulation of gene expression [69,70,71]. A well-studied P-responsive miRNA is miR399, which undergoes long-distance shoot-to-root allocation during the onset of P deficiency [69] and is suggested to mediate enhanced P uptake and translocation [72]. miR399 post-transcriptionally regulates PHO2 (PHOSPHATE 2), a ubiquitin-conjugating E2 enzyme that targets members of the PHT1 (PHOSPHATE TRANSPORTER 1) family for ubiquitin-mediated degradation [69,72,73,74].

Phosphate starvation and AM-symbiosis-related signaling networks interlink, and miRNAs are among the shared components. For example, the miR399-*PHO2* node-regulating P-homeostasis in non-mycorrhizal plants was shown to be acting in AM-colonized roots of *M. truncatula* [75]. Studies in different symbiotic plants have identified miRNAs that dually respond to P availability and AM fungal infection. For example, miR393, shown to restrict arbuscule development by targeting auxin receptors involved in arbuscule formation, is induced by low P-concentrations and repressed by AM [76]. The responsiveness of different miRNAs in the environmental P conditions and their roles in AM symbiosis are reviewed in [59]. A particularly interesting antagonistic role is reported for miR171 isoforms in *M. truncatula* AM symbiosis control. Several miR171 family members negatively regulate root invasion by AM fungi via post-transcriptional control of the GRAS-type transcription factor LOM (LOST MERISTEMS 1), a positive regulator of AM [77]. In contrast, miR171b, which specifically accumulates in arbuscule-containing plant cells, displays a mismatched cleavage site and prevents cleavage of *LOM1* transcripts by other members of the miR171 family [77].

*M. truncatula* miR171h (*L. japonicus* miR171c), which targets the GRAS-type transcription factor *NODULATION SIGNALLING PATHWAY2 (NSP2)* transcripts [78,79], accumulates under both N and P sufficiency and has been reported to be involved in both rhizobial nodulation and AM symbioses. NSP2 is essential for nodulation in legumes [80,81], and positively regulates AM fungal colonization [15]. It is further involved in strigolactone biosynthesis [82]. In line with its roles in symbiosis development, *M. truncatula* miR171h accumulation is not only nutrient-status-dependent, but also induced by myc-LCO and nod factor signaling during AM and nodulation symbioses [78,79,83]. Ectopic overexpression of pri-miR171h in *M. truncatula* roots resulted in reduced mycorrhizal root colonization and nodule numbers compared to controls, when plants were inoculated with AM fungi and rhizobia, respectively [83]. Thus miR171h seems to have a central role in integrating plant responses to the essential nutrients N and P, and the acquisition of these nutrients through symbiotic associations.

Several miRNAs have been reported to respond to rhizobial inoculation (Table 2, Figure 1) and are presumed to play roles during early stages of the symbiotic interaction, mostly by targeting transcripts of genes encoding transcription factors. Apart from miR171 family members, these include miR319d in common beans and miR172 in many plant species (reviewed in [84]). The sequencing of sRNA libraries from nodules alongside a degradome analysis identified several miRNA-target pairs that show activity in nodules. In soybeans, combined sRNA and degradome sequencing revealed miR167 targeting the 5’UTR of the nuclear cation channel CYCLOPS as well as miR393j-3p targeting of *ENOD93* (*EARLY NODULIN 93*) [85]. The overexpression of miR393 in soybean roots significantly reduced nodulation [85]. *M. truncatula* miR167 family members further target auxin response factors [86], and *L. japonicus* miR397 targets a Cu^2+^-containing LACCASE [79]. The regulation of some miRNAs has been linked to auxin and cytokinin action in the legume–rhizobium symbiosis, however there are only a few studies on this topic (reviewed in [84]). Interestingly, in line with an adaptation of conserved developmental mechanisms in the genetic regulation of symbiosis, *M. truncatula* miR166 has a dual role regulating root and nodule development. miR166 isoforms target *HD-ZIPIII* (*CLASS-III HOMEODOMAIN-LEUCINE ZIPPER*) genes, a family of transcription factors associated with nodule development, and overexpression of *MtMIR166* affected both nodule and lateral root numbers as well as vascular bundle development [87].

Over the last years, miR2111 has emerged as a key component of root nodulation control via the AON mechanism (discussed in Section 6 of the current article). miR2111 is a mobile signal undergoing shoot-to-root translocation. It accumulates in shoots under low N conditions and acts as a positive regulator of nodulation by targeting root-localized transcripts encoding the F-Box Kelch-repeat protein TML (TOO MUCH LOVE) [88], an inhibitor of rhizobial infection and nodulation [89,90].

A second miRNA implemented in AON is soybean miR172c [91,92]. miR172c strongly accumulates in the vicinity of rhizobial invasion and in nodules [91,93] and acts as a positive regulator of rhizobial infection and nodule formation through regulation of AP2/ERF transcription factor mRNAs [91,93]. In soybeans, the transcriptional repressor NNC1 (NODULE NUMBER CONTROL 1) is assumed to be the primary miR172 target [91,92]. NNC1 is a negative regulator of nodulation and was shown to bind to the promoters of the early nodulin genes *ENOD40-1* and *-2,* inhibiting their expression. NNC1 further interacts with NIN (NODULE INCEPTION), inhibiting the transcription of downstream genes encoding *Gm*RIC1 and *Gm*RIC2 peptides, linking it to AON. Using a *nark* loss-of-function mutant, it was shown that miR172c is negatively regulated by NARK, an observation providing additional evidence for the involvement of the miR172c-*NNC1* node in AON in soybeans [91,92].

**Table 2:** List of selected miRNAs responding to nitrogen (N), phosphorus (P) and microsymbionts (rhizobium and AM fungi).

	Stimuli	Organism	Influence	Tissue	Target	Refs
miR167	N-repressed	*A. thaliana*	local	root pericycle cells	*ARF8*	[65]
miR169	N-limitation repressed	*A. thaliana and B. napus*	systemic	shoots, roots, phloem sap	*NFYA5*	[67] [70]
miR398a	N-limitation and P-limitation repressed	*A. thaliana*				[70]
miR399	P-limitation induced	*A. thaliana* *and B. napus*	systemic	vascular tissues, phloem sap	*PHO2*	[70] [72]
miR2111	P-limitation induced N-repressed, rhizobium-repressed	*A. thaliana and B. napus* *L. japonicus*	systemic	phloem sap leaves phloem, phloem sap	*E3 ligase* *TML*	[76] [88]
miR397	nodulation-induced	*L. japonicus*	local and systemic	nodules, leaves	*LACCASE10*	[79]
miR171c	nodulation-induced	*L. japonicus*		nodules	*NSP2*	[79]
miR171h	expressed in high P and N, AM-repressed, nodulation-induced	*M. truncatula*		roots, arbuscule-containing cells, nodules	*NSP2*	[83]
miR171b	AM-specific	*M. truncatula*	local	colonized root cells	*LOM1*	[77]
miR393	low-P expressed, AM-repressed	*M. truncatula*	local	roots	*auxin receptors*	[76]
miR399	low P-induced, AM-induced	*M*. *truncatula*	systemic	leaves and roots	*PHO2*	[75]
miR166	nodulation induced	*M*. *truncatula*	local	vascular bundles, roots, nodules	*HD-ZIP III*	[87]
miR172c	rhizobium-induced, nod factors-induced	*G. max*	local	rhizobium-inoculated roots and nodules	*NNC1*	[91]
miR156b	rhizobium-repressed	*G. max*	local	roots	*GmSPL9d*	[94]

In a recent report, Yun et al. [94] showed that overexpression of miR156b in soybean roots resulted in reduced expression of *NINa*, *ENOD40-1* and *MIR172c*. The main target of miR156b is the *GmSPL9d* (*SQUAMOSA PROMOTER-BINDING LIKE 9d*) gene, a positive regulator of symbiosis that accumulates upon infection. *Gm*SPL9d affects the expression of *NINa*, *ENOD40-1* and *MIR172c* by direct promoter binding [94]. Similarly, in *L. japonicus*, ectopic overexpression of miR156a reduced nodulation and affected the expression of early nodulation genes such as *ENOD* genes, *NFR1*, *CYCLOPS* and *NSP1* [95]. These data suggest that the miR156-SPL node has a key regulatory role in nodulation by directly activating the expression of core genes in the early stages of nodulation signaling.

## 6. CEPs and CLEs and miR2111 Jointly Orchestrate Plant Responses to N and Rhizobia

AON controls rhizobial infection and nodule numbers to ensure a viable balance between ammonia uptake and carbohydrate as well as nutrient costs. This feedback loop has been well-described in different plant species and was shown to be systemic, involving CLE and CEP peptides as root-derived signals moving to the shoot through the xylem, and micro RNA miR2111 as well as CEPD peptides as shoot-derived, root-active signals navigating through the phloem [56,88]. miR2111 is mainly expressed in shoot tissues, more precisely in leaf vein phloem [88,96]. Leaf phloem expression was postulated as prerequisite for systemic mobility of small RNAs [97], and indeed, miR2111 was shown to translocate from shoot to root [88,96]. Shoot-derived miR2111 effectively reduces root transcript levels of *TML* via endonucleolytic cleavage [88].

The miR2111-*TML* node is responsive to both soil nitrate levels and rhizobial signaling, suggesting a role in balancing nodulation symbiosis with N availability. Nitrate fertilization or rhizobial inoculation led to decreased miR2111 levels, and accordingly, *TML* transcript abundance increased [56,88].

miR2111 expression depends on two peptide receptors, the LRR-RLKs *Lj*HAR1/*Mt*SUNN/*Gm*NARK and *Mt*CRA2 [37,56,88,96]. Both factors are expressed in the whole plant, however the regulation of symbiosis is mainly achieved by the shoot fraction. HAR1, a negative regulator of symbiosis, represses miR2111 levels in rhizobially infected plants, resulting in *TML* transcript accumulation and restriction of further infections [88,96]. The second regulator of miR2111, CRA2, is a positive regulator of symbiosis and promotes miR2111 accumulation under low N conditions [56]. The antagonistic regulation of infection through miR2111 underlines the biological relevance of this node. For the plant, both miR2111 promotion and repression, and a fast switch of these states, seem equally important, allowing the plant to quickly change from a susceptible status welcoming infection to restriction of the latter.

Consistent with the divergent effects of activated HAR1/SUNN/NARK and CRA2 on miR2111 regulation, these two shoot receptors differ in the groups of peptide ligands they perceive. HAR1/SUNN/NARK interacts with CLE peptides [29,33], and several studies suggest that the receptor regulates miR2111 depending on root-derived CLE peptide perception [37,88,96]. For example, nitrate induction of *MtCLE35* coincided with reduced miR2111 levels and, consistently, an accumulation of *MtTML2* transcripts downstream of the SUNN receptor [37]. CRA2, in contrast, perceives CEP peptide ligands, positively regulating miR2111 depending on the presence of CEPs [56]. Overexpression of *MtCEP1*, for example, resulted in increased miR2111 abundance and reduced transcript levels of both *M. truncatula TML1* and *TML2* in roots. This was dependent on CRA2, as those effects were not apparent in *cra2* mutants [56]. Both classes of peptides possess several members regulated by N and/or symbiosis signaling (see Section 3 of the current article) (Table 1, Figure 1). The combined results indicate that a multitude of CEP and CLE peptide signals triggering divergent responses converge in the miR2111-*TML* regulon, shaping a model of AON as a complex, multilayered network that dynamically integrates infection and symbiosis development with plant nutritional status and needs.

## 7. CLE Peptide Involvement in P-Dependent Control of Nodulation

P supply is well known to positively correlate with nodulation and symbiotic N fixation in legumes [98,99], and consistently, nodule fresh weight and activity are sensitive to P deficiency [100]. A study in the actinorhizal plant *Alnus incana* showed that a high phosphate concentration can reverse the nitrate-induced inhibition of nodulation, leading to an increase of nodules. The positive effect of P on nodule numbers was found to be systemically regulated and independent of overall plant growth and development [101]. In common beans, P deficiency reduced the numbers of the bacterially induced root hair deformations during the initial steps of rhizobial infection [102]. Although a negative effect of P deficiency on nodulation has been clearly documented, the underlying molecular mechanisms were unknown until recently.

In the roots of common beans, P deficiency induced the expression of genes encoding the AON-related root-to-shoot signals RIC1 and RIC2 in the absence of symbiosis [48] (Table 1). Moreover, it was shown that, under P deficiency, RIC1 and RIC2 led to a systemic restriction of nodulation, through the HAR1/SUNN/NARK receptor in both common beans and soybeans. This effect seems to be mediated by TML, as *TML* transcripts accumulated in the roots of both plants [48]. These data suggest that CLE peptides negatively regulate nodule formation under P deficiency conditions via the AON genetic network.

## 8. Conclusions

Plants have adopted different strategies to control nutrient homeostasis and overcome nutrient limitation in their environment, such as the adaptation of root system architecture and the establishment of root symbiotic relationships.

The molecular basis of these response systems has been the subject of intense interest by the scientific community in the light of reducing dependence on inorganic fertilizers while securing global food supplies. Studies in model plants revealed conserved processes that ensure survival and productivity under nutrient deprivation, and there is an increasing host of knowledge on how plants cope with fluctuations in the availability of important nutrients such as N and P in the soil. However, more research to this field is not only important for transferring the knowledge acquired in model systems to a wider range of species including crop plants, but also to grasp the relevance of these processes in natural communities, and in adapting plant populations to increasingly challenging environmental conditions in the face of climate change.

## Data Availability

Not applicable.

## References

[1] Udvardi M., Poole P.S. (2013). Transport and Metabolism in Legume-Rhizobia Symbioses. Annu. Rev. Plant Biol..

[2] Patriarca E.J., Tatè R., Iaccarino M. (2002). Key Role of Bacterial NH_4_^+^ Metabolism in Rhizobium-Plant Symbiosis. Microbiol. Mol. Biol. Rev..

[3] Kaiser B.N., Finnegan P.M., Tyerman S.D., Whitehead L.F., Bergersen F.J., Day D.A., Udvardi M.K. (1998). Characterization of an Ammonium Transport Protein from the Peribacteroid Membrane of Soybean Nodules. Science.

[4] Salvemini F., Marini A., Riccio A., Patriarca E.J., Chiurazzi M. (2001). Functional Characterization of an Ammonium Transporter Gene from *Lotus japonicus*. Gene.

[5] Maxwell C.A., Hartwig U.A., Joseph C.M., Phillips D.A. (1989). A Chalcone and Two Related Flavonoids Released from Alfalfa Roots Induce Nod Genes of Rhizobium Meliloti. Plant Physiol..

[6] Long S.R., Staskawicz B.J. (1993). Prokaryotic Plant Parasites. Cell.

[7] Dénarié J., Debellé F., Promé J.C. (1996). Rhizobium Lipo-Chitooligosaccharide Nodulation Factors: Signaling Molecules Mediating Recognition and Morphogenesis. Annu. Rev. Biochem..

[8] Oldroyd G.E.D., Murray J.D., Poole P.S., Downie J.A. (2011). The Rules of Engagement in the Legume-Rhizobial Symbiosis. Annu. Rev. Genet..

[9] Harrison M.J., van Buuren M.L. (1995). A Phosphate Transporter from the Mycorrhizal Fungus Glomus Versiforme. Nature.

[10] Hijikata N., Murase M., Tani C., Ohtomo R., Osaki M., Ezawa T. (2010). Polyphosphate Has a Central Role in the Rapid and Massive Accumulation of Phosphorus in Extraradical Mycelium of an Arbuscular Mycorrhizal Fungus. New Phytol..

[11] López-Pedrosa A., González-Guerrero M., Valderas A., Azcón-Aguilar C., Ferrol N. (2006). GintAMT1 Encodes a Functional High-Affinity Ammonium Transporter That Is Expressed in the Extraradical Mycelium of Glomus Intraradices. Fungal Genet. Biol..

[12] Cappellazzo G., Lanfranco L., Fitz M., Wipf D., Bonfante P. (2008). Characterization of an Amino Acid Permease from the Endomycorrhizal Fungus Glomus Mosseae. Plant Physiol..

[13] MacLean A.M., Bravo A., Harrison M.J. (2017). Plant Signaling and Metabolic Pathways Enabling Arbuscular Mycorrhizal Symbiosis. Plant Cell.

[14] Akiyama K., Matsuzaki K., Hayashi H. (2005). Plant Sesquiterpenes Induce Hyphal Branching in Arbuscular Mycorrhizal Fungi. Nature.

[15] Maillet F., Poinsot V., André O., Puech-Pagès V., Haouy A., Gueunier M., Cromer L., Giraudet D., Formey D., Niebel A. (2011). Fungal Lipochitooligosaccharide Symbiotic Signals in Arbuscular Mycorrhiza. Nature.

[16] Genre A., Chabaud M., Balzergue C., Puech-Pagès V., Novero M., Rey T., Fournier J., Rochange S., Bécard G., Bonfante P. (2013). Short-Chain Chitin Oligomers from Arbuscular Mycorrhizal Fungi Trigger Nuclear Ca^2+^ Spiking in *Medicago truncatula* Roots and Their Production Is Enhanced by Strigolactone. New Phytol..

[17] Nagahashi G., Douds D.D. (1997). Appressorium Formation by AM Fungi on Isolated Cell Walls of Carrot Roots. New Phytol..

[18] Bonfante P., Genre A. (2010). Mechanisms Underlying Beneficial Plant–Fungus Interactions in Mycorrhizal Symbiosis. Nat. Commun..

[19] Gutjahr C., Parniske M. (2017). Cell Biology: Control of Partner Lifetime in a Plant–Fungus Relationship. Curr. Biol..

[20] de Bang T.C., Lay K.S., Scheible W.-R., Takahashi H. (2017). Small Peptide Signaling Pathways Modulating Macronutrient Utilization in Plants. Curr. Opin. Plant Biol..

[21] de Bang T.C., Lundquist P.K., Dai X., Boschiero C., Zhuang Z., Pant P., Torres-Jerez I., Roy S., Nogales J., Veerappan V. (2017). Genome-Wide Identification of Medicago Peptides Involved in Macronutrient Responses and Nodulation. Plant Physiol..

[22] Betsuyaku S., Sawa S., Yamada M. (2011). The Function of the CLE Peptides in Plant Development and Plant-Microbe Interactions. Arab. Book.

[23] Yamaguchi Y.L., Ishida T., Sawa S. (2016). CLE Peptides and Their Signaling Pathways in Plant Development. J. Exp. Bot..

[24] Araya T., Miyamoto M., Wibowo J., Suzuki A., Kojima S., Tsuchiya Y.N., Sawa S., Fukuda H., von Wirén N., Takahashi H. (2014). CLE-CLAVATA1 Peptide-Receptor Signaling Module Regulates the Expansion of Plant Root Systems in a Nitrogen-Dependent Manner. Proc. Natl. Acad. Sci. USA.

[25] Chaulagain D., Frugoli J. (2021). The Regulation of Nodule Number in Legumes Is a Balance of Three Signal Transduction Pathways. Int. J. Mol. Sci..

[26] Okamoto S., Ohnishi E., Sato S., Takahashi H., Nakazono M., Tabata S., Kawaguchi M. (2009). Nod Factor/Nitrate-Induced CLE Genes That Drive HAR1-Mediated Systemic Regulation of Nodulation. Plant Cell Physiol..

[27] Nishida H., Handa Y., Tanaka S., Suzaki T., Kawaguchi M. (2016). Expression of the CLE-RS3 Gene Suppresses Root Nodulation in *Lotus japonicus*. J. Plant Res..

[28] Mortier V., Den Herder G., Whitford R., Van de Velde W., Rombauts S., D’Haeseleer K., Holsters M., Goormachtig S. (2010). CLE Peptides Control *Medicago truncatula* Nodulation Locally and Systemically. Plant Physiol..

[29] Lebedeva M., Azarakhsh M., Yashenkova Y., Lutova L. (2020). Nitrate-Induced CLE Peptide Systemically Inhibits Nodulation in *Medicago truncatula*. Plants.

[30] Mens C., Hastwell A.H., Su H., Gresshoff P.M., Mathesius U., Ferguson B.J. (2021). Characterisation of *Medicago truncatula* CLE34 and CLE35 in Nitrate and Rhizobia Regulation of Nodulation. New Phytol..

[31] Reid D.E., Ferguson B.J., Gresshoff P.M. (2011). Inoculation- and Nitrate-Induced CLE Peptides of Soybean Control NARK-Dependent Nodule Formation. Mol. Plant-Microbe Interact..

[32] Ferguson B.J., Li D., Hastwell A.H., Reid D.E., Li Y., Jackson S.A., Gresshoff P.M. (2014). The Soybean (*Glycine max*) Nodulation-Suppressive CLE Peptide, GmRIC1, Functions Interspecifically in Common White Bean (*Phaseolus vulgaris*), but Not in a Supernodulating Line Mutated in the Receptor PvNARK. Plant Biotechnol. J..

[33] Okamoto S., Shinohara H., Mori T., Matsubayashi Y., Kawaguchi M. (2013). Root-Derived CLE Glycopeptides Control Nodulation by Direct Binding to HAR1 Receptor Kinase. Nat. Commun..

[34] Krusell L., Madsen L.H., Sato S., Aubert G., Genua A., Szczyglowski K., Duc G., Kaneko T., Tabata S., de Bruijn F. (2002). Shoot Control of Root Development and Nodulation Is Mediated by a Receptor-like Kinase. Nature.

[35] Nishimura R., Hayashi M., Wu G.-J., Kouchi H., Imaizumi-Anraku H., Murakami Y., Kawasaki S., Akao S., Ohmori M., Nagasawa M. (2002). HAR1 Mediates Systemic Regulation of Symbiotic Organ Development. Nature.

[36] Searle I.R., Men A.E., Laniya T.S., Buzas D.M., Iturbe-Ormaetxe I., Carroll B.J., Gresshoff P.M. (2003). Long-Distance Signaling in Nodulation Directed by a CLAVATA1-like Receptor Kinase. Science.

[37] Moreau C., Gautrat P., Frugier F. (2021). Nitrate-Induced CLE35 Signaling Peptides Inhibit Nodulation through the SUNN Receptor and MiR2111 Repression. Plant Physiol..

[38] Ohyama K., Shinohara H., Ogawa-Ohnishi M., Matsubayashi Y. (2009). A Glycopeptide Regulating Stem Cell Fate in Arabidopsis Thaliana. Nat. Chem. Biol..

[39] Ogawa-Ohnishi M., Matsushita W., Matsubayashi Y. (2013). Identification of Three Hydroxyproline O-Arabinosyltransferases in Arabidopsis Thaliana. Nat. Chem. Biol..

[40] Kassaw T., Nowak S., Schnabel E., Frugoli J. (2017). ROOT DETERMINED NODULATION1 Is Required for M. Truncatula CLE12, But Not CLE13, Peptide Signaling through the SUNN Receptor Kinase. Plant Physiol..

[41] Imin N., Patel N., Corcilius L., Payne R.J., Djordjevic M.A. (2018). CLE Peptide Tri-Arabinosylation and Peptide Domain Sequence Composition Are Essential for SUNN-Dependent Autoregulation of Nodulation in *Medicago truncatula*. New Phytol..

[42] Hastwell A.H., Corcilius L., Williams J.T., Gresshoff P.M., Payne R.J., Ferguson B.J. (2019). Triarabinosylation Is Required for Nodulation-Suppressive CLE Peptides to Systemically Inhibit Nodulation in Pisum Sativum. Plant Cell Environ..

[43] Yoro E., Nishida H., Ogawa-Ohnishi M., Yoshida C., Suzaki T., Matsubayashi Y., Kawaguchi M. (2019). PLENTY, a Hydroxyproline O-Arabinosyltransferase, Negatively Regulates Root Nodule Symbiosis in *Lotus japonicus*. J. Exp. Bot..

[44] Funayama-Noguchi S., Noguchi K., Yoshida C., Kawaguchi M. (2011). Two CLE Genes Are Induced by Phosphate in Roots of *Lotus japonicus*. J. Plant Res..

[45] Laffont C., Ivanovici A., Gautrat P., Brault M., Djordjevic M.A., Frugier F. (2020). The NIN Transcription Factor Coordinates CEP and CLE Signaling Peptides That Regulate Nodulation Antagonistically. Nat. Commun..

[46] Müller L.M., Flokova K., Schnabel E., Sun X., Fei Z., Frugoli J., Bouwmeester H.J., Harrison M.J. (2019). A CLE-SUNN Module Regulates Strigolactone Content and Fungal Colonization in Arbuscular Mycorrhiza. Nat. Plants.

[47] Karlo M., Boschiero C., Landerslev K.G., Blanco G.S., Wen J., Mysore K.S., Dai X., Zhao P.X., de Bang T.C. (2020). The CLE53-SUNN Genetic Pathway Negatively Regulates Arbuscular Mycorrhiza Root Colonization in *Medicago truncatula*. J. Exp. Bot..

[48] Isidra-Arellano M.C., Pozas-Rodríguez E.A., Del Rocío Reyero-Saavedra M., Arroyo-Canales J., Ferrer-Orgaz S., Del Socorro Sánchez-Correa M., Cardenas L., Covarrubias A.A., Valdés-López O. (2020). Inhibition of Legume Nodulation by Pi Deficiency Is Dependent on the Autoregulation of Nodulation (AON) Pathway. Plant J..

[49] Tabata R., Sumida K., Yoshii T., Ohyama K., Shinohara H., Matsubayashi Y. (2014). Perception of Root-Derived Peptides by Shoot LRR-RKs Mediates Systemic N-Demand Signaling. Science.

[50] Imin N., Mohd-Radzman N.A., Ogilvie H.A., Djordjevic M.A. (2013). The Peptide-Encoding CEP1 Gene Modulates Lateral Root and Nodule Numbers in *Medicago truncatula*. J. Exp. Bot..

[51] Laffont C., Huault E., Gautrat P., Endre G., Kalo P., Bourion V., Duc G., Frugier F. (2019). Independent Regulation of Symbiotic Nodulation by the SUNN Negative and CRA2 Positive Systemic Pathways. Plant Physiol..

[52] Zhu F., Ye Q., Chen H., Dong J., Wang T. (2021). Multigene Editing Reveals That MtCEP1/2/12 Redundantly Control Lateral Root and Nodule Number in *Medicago truncatula*. J. Exp. Bot..

[53] Hsieh Y.-H., Wei Y.-H., Lo J.-C., Pan H.-Y., Yang S.-Y. (2022). Arbuscular Mycorrhizal Symbiosis Enhances Tomato Lateral Root Formation by Modulating CEP2 Peptide Expression. New Phytol..

[54] Ohyama K., Ogawa M., Matsubayashi Y. (2008). Identification of a Biologically Active, Small, Secreted Peptide in Arabidopsis by in Silico Gene Screening, Followed by LC-MS-Based Structure Analysis. Plant J..

[55] Huault E., Laffont C., Wen J., Mysore K.S., Ratet P., Duc G., Frugier F. (2014). Local and Systemic Regulation of Plant Root System Architecture and Symbiotic Nodulation by a Receptor-like Kinase. PLoS Genet..

[56] Gautrat P., Laffont C., Frugier F. (2020). Compact Root Architecture 2 Promotes Root Competence for Nodulation through the MiR2111 Systemic Effector. Curr. Biol..

[57] Roy S., Müller L.M. (2022). A Rulebook for Peptide Control of Legume-Microbe Endosymbioses. Trends Plant Sci.

[58] Breuillin F., Schramm J., Hajirezaei M., Ahkami A., Favre P., Druege U., Hause B., Bucher M., Kretzschmar T., Bossolini E. (2010). Phosphate Systemically Inhibits Development of Arbuscular Mycorrhiza in *Petunia hybrida* and Represses Genes Involved in Mycorrhizal Functioning. Plant J..

[59] Müller L.M., Harrison M.J. (2019). Phytohormones, MiRNAs, and Peptide Signals Integrate Plant Phosphorus Status with Arbuscular Mycorrhizal Symbiosis. Curr. Opin. Plant Biol..

[60] Meixner C., Ludwig-Müller J., Miersch O., Gresshoff P., Staehelin C., Vierheilig H. (2005). Lack of Mycorrhizal Autoregulation and Phytohormonal Changes in the Supernodulating Soybean Mutant Nts1007. Planta.

[61] Wang C., Reid J.B., Foo E. (2018). The Art of Self-Control—Autoregulation of Plant–Microbe Symbioses. Front. Plant Sci..

[62] Handa Y., Nishide H., Takeda N., Suzuki Y., Kawaguchi M., Saito K. (2015). RNA-Seq Transcriptional Profiling of an Arbuscular Mycorrhiza Provides Insights into Regulated and Coordinated Gene Expression in *Lotus japonicus* and *Rhizophagus irregularis*. Plant Cell Physiol..

[63] van Zeijl A., Liu W., Xiao T.T., Kohlen W., Yang W.-C., Bisseling T., Geurts R. (2015). The Strigolactone Biosynthesis Gene DWARF27 Is Co-Opted in Rhizobium Symbiosis. BMC Plant Biol..

[64] Liang G., He H., Yu D. (2012). Identification of Nitrogen Starvation-Responsive MicroRNAs in Arabidopsis Thaliana. PLoS ONE.

[65] Gifford M.L., Dean A., Gutierrez R.A., Coruzzi G.M., Birnbaum K.D. (2008). Cell-Specific Nitrogen Responses Mediate Developmental Plasticity. Proc. Natl. Acad. Sci. USA.

[66] Vidal E.A., Araus V., Lu C., Parry G., Green P.J., Coruzzi G.M., Gutiérrez R.A. (2010). Nitrate-Responsive MiR393/AFB3 Regulatory Module Controls Root System Architecture in Arabidopsis Thaliana. Proc. Natl. Acad. Sci. USA.

[67] Zhao M., Ding H., Zhu J.-K., Zhang F., Li W.-X. (2011). Involvement of MiR169 in the Nitrogen-Starvation Responses in Arabidopsis. New Phytol..

[68] Fukuda M., Fujiwara T., Nishida S. (2020). Roles of Non-Coding RNAs in Response to Nitrogen Availability in Plants. Int. J. Mol. Sci..

[69] Pant B.D., Buhtz A., Kehr J., Scheible W.-R. (2008). MicroRNA399 Is a Long-Distance Signal for the Regulation of Plant Phosphate Homeostasis. Plant J..

[70] Pant B.D., Musialak-Lange M., Nuc P., May P., Buhtz A., Kehr J., Walther D., Scheible W.-R. (2009). Identification of Nutrient-Responsive Arabidopsis and Rapeseed MicroRNAs by Comprehensive Real-Time Polymerase Chain Reaction Profiling and Small RNA Sequencing. Plant Physiol..

[71] Huen A., Bally J., Smith P. (2018). Identification and Characterisation of MicroRNAs and Their Target Genes in Phosphate-Starved Nicotiana Benthamiana by Small RNA Deep Sequencing and 5′RACE Analysis. BMC Genom..

[72] Lin S.-I., Chiang S.-F., Lin W.-Y., Chen J.-W., Tseng C.-Y., Wu P.-C., Chiou T.-J. (2008). Regulatory Network of MicroRNA399 and PHO2 by Systemic Signaling. Plant Physiol..

[73] Aung K., Lin S.-I., Wu C.-C., Huang Y.-T., Su C.-L., Chiou T.-J. (2006). Pho2, a Phosphate Overaccumulator, Is Caused by a Nonsense Mutation in a MicroRNA399 Target Gene. Plant Physiol..

[74] Huang T.-K., Han C.-L., Lin S.-I., Chen Y.-J., Tsai Y.-C., Chen Y.-R., Chen J.-W., Lin W.-Y., Chen P.-M., Liu T.-Y. (2013). Identification of Downstream Components of Ubiquitin-Conjugating Enzyme PHOSPHATE2 by Quantitative Membrane Proteomics in Arabidopsis Roots. Plant Cell.

[75] Branscheid A., Sieh D., Pant B.D., May P., Devers E.A., Elkrog A., Schauser L., Scheible W.-R., Krajinski F. (2010). Expression Pattern Suggests a Role of MiR399 in the Regulation of the Cellular Response to Local Pi Increase during Arbuscular Mycorrhizal Symbiosis. Mol. Plant-Microbe Interact..

[76] Etemadi M., Gutjahr C., Couzigou J.-M., Zouine M., Lauressergues D., Timmers A., Audran C., Bouzayen M., Bécard G., Combier J.-P. (2014). Auxin Perception Is Required for Arbuscule Development in Arbuscular Mycorrhizal Symbiosis. Plant Physiol..

[77] Couzigou J.-M., Lauressergues D., André O., Gutjahr C., Guillotin B., Bécard G., Combier J.-P. (2017). Positive Gene Regulation by a Natural Protective MiRNA Enables Arbuscular Mycorrhizal Symbiosis. Cell Host Microbe.

[78] Lauressergues D., Delaux P.-M., Formey D., Lelandais-Brière C., Fort S., Cottaz S., Bécard G., Niebel A., Roux C., Combier J.-P. (2012). The MicroRNA MiR171h Modulates Arbuscular Mycorrhizal Colonization of *Medicago truncatula* by Targeting NSP2. Plant J..

[79] De Luis A., Markmann K., Cognat V., Holt D.B., Charpentier M., Parniske M., Stougaard J., Voinnet O. (2012). Two MicroRNAs Linked to Nodule Infection and Nitrogen-Fixing Ability in the Legume *Lotus japonicus*. Plant Physiol..

[80] Kaló P., Gleason C., Edwards A., Marsh J., Mitra R.M., Hirsch S., Jakab J., Sims S., Long S.R., Rogers J. (2005). Nodulation Signaling in Legumes Requires NSP2, a Member of the GRAS Family of Transcriptional Regulators. Science.

[81] Heckmann A.B., Lombardo F., Miwa H., Perry J.A., Bunnewell S., Parniske M., Wang T.L., Downie J.A. (2006). *Lotus japonicus* Nodulation Requires Two GRAS Domain Regulators, One of Which Is Functionally Conserved in a Non-Legume. Plant Physiol..

[82] Liu W., Kohlen W., Lillo A., Op den Camp R., Ivanov S., Hartog M., Limpens E., Jamil M., Smaczniak C., Kaufmann K. (2011). Strigolactone Biosynthesis in *Medicago truncatula* and Rice Requires the Symbiotic GRAS-Type Transcription Factors NSP1 and NSP2. Plant Cell.

[83] Hofferek V., Mendrinna A., Gaude N., Krajinski F., Devers E.A. (2014). MiR171h Restricts Root Symbioses and Shows like Its Target NSP2 a Complex Transcriptional Regulation in *Medicago truncatula*. BMC Plant Biol..

[84] Hoang N.T., Tóth K., Stacey G. (2020). The Role of MicroRNAs in the Legume-Rhizobium Nitrogen-Fixing Symbiosis. J. Exp. Bot..

[85] Yan Z., Hossain M.S., Arikit S., Valdés-López O., Zhai J., Wang J., Libault M., Ji T., Qiu L., Meyers B.C. (2015). Identification of MicroRNAs and Their MRNA Targets during Soybean Nodule Development: Functional Analysis of the Role of MiR393j-3p in Soybean Nodulation. New Phytol..

[86] Lelandais-Brière C., Naya L., Sallet E., Calenge F., Frugier F., Hartmann C., Gouzy J., Crespi M. (2009). Genome-Wide *Medicago truncatula* Small RNA Analysis Revealed Novel MicroRNAs and Isoforms Differentially Regulated in Roots and Nodules. Plant Cell.

[87] Boualem A., Laporte P., Jovanovic M., Laffont C., Plet J., Combier J.-P., Niebel A., Crespi M., Frugier F. (2008). MicroRNA166 Controls Root and Nodule Development in *Medicago truncatula*. Plant J..

[88] Tsikou D., Yan Z., Holt D.B., Abel N.B., Reid D.E., Madsen L.H., Bhasin H., Sexauer M., Stougaard J., Markmann K. (2018). Systemic Control of Legume Susceptibility to Rhizobial Infection by a Mobile MicroRNA. Science.

[89] Magori S., Oka-Kira E., Shibata S., Umehara Y., Kouchi H., Hase Y., Tanaka A., Sato S., Tabata S., Kawaguchi M. (2009). Too Much Love, a Root Regulator Associated with the Long-Distance Control of Nodulation in *Lotus japonicus*. Mol. Plant-Microbe Interact..

[90] Takahara M., Magori S., Soyano T., Okamoto S., Yoshida C., Yano K., Sato S., Tabata S., Yamaguchi K., Shigenobu S. (2013). Too Much Love, a Novel Kelch Repeat-Containing F-Box Protein, Functions in the Long-Distance Regulation of the Legume–*Rhizobium* Symbiosis. Plant Cell Physiol..

[91] Wang Y., Wang L., Zou Y., Chen L., Cai Z., Zhang S., Zhao F., Tian Y., Jiang Q., Ferguson B.J. (2014). Soybean MiR172c Targets the Repressive AP2 Transcription Factor NNC1 to Activate ENOD40 Expression and Regulate Nodule Initiation. Plant Cell.

[92] Wang L., Sun Z., Su C., Wang Y., Yan Q., Chen J., Ott T., Li X. (2019). A GmNINa-MiR172c-NNC1 Regulatory Network Coordinates the Nodulation and Autoregulation of Nodulation Pathways in Soybean. Mol. Plant.

[93] Holt D.B., Gupta V., Meyer D., Abel N.B., Andersen S.U., Stougaard J., Markmann K. (2015). Micro RNA 172 (MiR172) Signals Epidermal Infection and Is Expressed in Cells Primed for Bacterial Invasion in *Lotus japonicus* Roots and Nodules. New Phytol..

[94] Yun J., Sun Z., Jiang Q., Wang Y., Wang C., Luo Y., Zhang F., Li X. (2022). The MiR156b-GmSPL9d Module Modulates Nodulation by Targeting Multiple Core Nodulation Genes in Soybean. New Phytol..

[95] Wang Y., Wang Z., Amyot L., Tian L., Xu Z., Gruber M.Y., Hannoufa A. (2015). Ectopic Expression of MiR156 Represses Nodulation and Causes Morphological and Developmental Changes in *Lotus japonicus*. Mol. Genet. Genom..

[96] Okuma N., Soyano T., Suzaki T., Kawaguchi M. (2020). MIR2111-5 Locus and Shoot-Accumulated Mature MiR2111 Systemically Enhance Nodulation Depending on HAR1 in *Lotus japonicus*. Nat. Commun..

[97] Skopelitis D.S., Hill K., Klesen S., Marco C.F., von Born P., Chitwood D.H., Timmermans M.C.P. (2018). Gating of MiRNA Movement at Defined Cell-Cell Interfaces Governs Their Impact as Positional Signals. Nat. Commun..

[98] Jakobsen I. (1985). The Role of Phosphorus in Nitrogen Fixation by Young Pea Plants (*Pisum sativum*). Physiol. Plant..

[99] Kuang R.-B., Liao H., Yan X.-L., Dong Y.-S. (2005). Phosphorus and Nitrogen Interactions in Field-Grown Soybean as Related to Genetic Attributes of Root Morphological and Nodular Traits. J. Integr. Plant Biol..

[100] Divito G.A., Sadras V.O. (2014). How Do Phosphorus, Potassium and Sulphur Affect Plant Growth and Biological Nitrogen Fixation in Crop and Pasture Legumes? A Meta-Analysis. Field Crops Res..

[101] Gentili F., Huss-Danell K. (2003). Local and Systemic Effects of Phosphorus and Nitrogen on Nodulation and Nodule Function in *Alnus incana*. J. Exp. Bot..

[102] Isidra-Arellano M.C., Reyero-Saavedra M.D.R., Sánchez-Correa M.D.S., Pingault L., Sen S., Joshi T., Girard L., Castro-Guerrero N.A., Mendoza-Cozatl D.G., Libault M. (2018). Phosphate Deficiency Negatively Affects Early Steps of the Symbiosis between Common Bean and Rhizobia. Genes.

